# Severe tachycardia induced by dopamine in an elderly patient with renal insufficiency: A case report and literature review

**DOI:** 10.1097/MD.0000000000046553

**Published:** 2025-12-19

**Authors:** Hui Xu

**Affiliations:** a Gaozhao Street Community Health Service Center, Jiaxing, Zhejiang Province, China.

**Keywords:** dopamine, heart failure, pharmaceutical care, renal insufficiency, tachycardia

## Abstract

**Rationale:**

Dopamine remains a widely used inotrope in acute heart failure and cardiorenal syndrome; however, its administration carries a substantial risk in vulnerable patients. Current evidence suggests that conventional dosing strategies may lead to adverse cardiovascular outcomes. Here, we report a case highlighting the importance of conservative dopamine titration and structured monitoring to optimize both efficacy and safety.

**Patient concerns:**

A high-risk patient with acute decompensated heart failure presented with progressive dyspnea, oliguria, and hypotension. The patient exhibited significant hemodynamic instability, raising concerns about the use of dopamine as an inotropic agent.

**Diagnoses:**

The clinical evaluation supported a diagnosis of acute decompensated heart failure with concurrent cardiorenal syndrome based on reduced urine output, elevated creatinine levels, pulmonary congestion, and echocardiographic findings.

**Interventions:**

Dopamine infusion was initiated at a conservative starting dose (≤1.5 μg/kg/min), During subsequent cautious upward titration, the patient developed symptomatic tachyarrhythmia, prompting immediate discontinuation of the infusion. Patient management was guided by a structured monitoring pathway, incorporating bedside clinical assessment, pharmacist input, and electronic clinical decision support.

**Outcomes:**

The initial dopamine infusion improved urine output and stabilized systemic blood pressure. However, during dose titration, the patient developed a symptomatic tachyarrhythmia with ventricular rates reaching 180 bpm, classified as a “probable” adverse drug reaction. Upon discontinuation of dopamine and with careful hemodynamic management, the arrhythmia resolved. The heart rate subsequently decreased from a pretreatment baseline of 106 beats/min to a safer range, underscoring the necessity of vigilant monitoring even with conservative initial dosing.

**Lessons:**

This case emphasizes that dopamine therapy should not follow a “one-size-fits-all” model. Conservative dosing, multidisciplinary monitoring, and the use of decision support tools can reduce medication errors and improve safety. Personalized inotrope strategies are essential in patients with high-risk heart failure patients.

## 1. Introduction

Dopamine continues to serve as a first-line inotrope and vasopressor in acute decompensated heart failure (ADHF) and cardiogenic shock. However, growing clinical experience underscores the persistent and often underrecognized safety concerns associated with its use. Although current guidelines support dopamine for its dose-dependent enhancement of cardiac output and blood pressure,^[1]^ a reappraisal of its pharmacologic basis is urgently needed, especially in medically complex patients. So-called “renal-dose” dopamine, intended to promote perfusion via dopaminergic receptors,^[2]^ demonstrates inconsistent and transient benefits in practice, casting doubt on its reliability. Moreover, intermediate to high doses, while augmenting contractility through β1-adrenergic stimulation, introduce substantial chronotropic and proarrhythmic risks that frequently outweigh the therapeutic benefits.^[3]^

Compounding these concerns is the shifting landscape of ADHF, which predominantly affects older individuals with numerous comorbidities. The triad of chronic kidney disease (CKD), type 2 diabetes, and cardiovascular disease, increasingly conceptualized as cardiorenal-metabolic syndrome, identifies a high-risk population that is particularly susceptible to adverse effects of dopamine.^[4]^ In these patients, tachyarrhythmias should be viewed not as incidental side effects, but as critical events that can induce ischemia and precipitate hemodynamic collapse.^[3]^ The risk is most pronounced among elderly patients with preexisting structural heart disease and multisystem impairment,^[5]^ and is further amplified by CKD-related electrophysiological disturbances.^[6]^ These insights argue for a fundamental shift away from one-size-fits-all dosing protocols toward individualized therapeutic strategies. There is an urgent need to embrace alternative approaches that provide hemodynamic support without incurring excessive proarrhythmic liability, thereby improving the outcomes in this vulnerable and evolving patient population.

The expanding utilization of angiotensin receptor-neprilysin inhibitors (ARNI) in heart failure with reduced ejection fraction introduces additional pharmacological complexity. By fundamentally altering the neurohormonal equilibrium,^[7]^ ARNI therapy might modulate the pharmacodynamic response to exogenous catecholamines, a potential interaction requiring further mechanistic clarification.^[8]^ The present case of life-threatening tachyarrhythmia in a septuagenarian with multiple risk determinants underscores 2 critical imperatives: thorough reevaluation of conventional dopamine dosing paradigms,^[9]^ and implementation of structured monitoring protocols for high-risk populations.^[10]^

Our observations align with the emerging recognition of pharmacotherapeutic vulnerabilities in cardiorenal metabolic syndrome. As demonstrated, conventional dopamine dosing requires downward adjustment (≤1.5 μg/kg/min) in elderly patients with estimated glomerular filtration rate (eGFR) < 30 mL/min/1.73 m^2^, particularly those receiving ARNI therapy. Continuous telemetry monitoring should be mandated during infusion, with escalation thresholds triggering immediate multidisciplinary review.

## 2. Case presentation

A 75-year-old male with a history of dilated cardiomyopathy (diagnosed 10 years prior), persistent atrial fibrillation, hypertension, type 2 diabetes mellitus, and chronic kidney disease was admitted for the evaluation of acute decompensated heart failure. His symptoms, which worsened over the preceding 10 days, included dyspnea, orthopnea, and peripheral edema following an upper respiratory tract infection. The patient’s outpatient medications included sacubitril/valsartan, furosemide, isosorbide mononitrate, and dapagliflozin.

On physical examination, the patient was hypotensive (blood pressure, 92/54 mm Hg) and exhibited facial edema, bibasilar crackles, and significant peripheral edema, consistent with New York Heart Association class IV heart failure. Initial laboratory studies revealed hyperkalemia (serum potassium 6.67 mmol/L), hyponatremia (132.8 mmol/L), and severe renal impairment, as indicated by a serum creatinine level of 240 μmol/L (eGFR 21.9 mL/min/1.73 m^2^, calculated using the CKD-EPI formula) and a blood urea nitrogen level of 21.39 mmol/L. The N-terminal pro-B-type natriuretic peptide was elevated at 669.90 pg/mL. Electrocardiography confirmed atrial fibrillation with a mean ventricular rate of 96 bpm and a left bundle branch block. Transthoracic echocardiography demonstrated global cardiac enlargement and a severely reduced left ventricular ejection fraction of 38%.

During hospitalization, dopamine infusion was initiated at 1.0 μg/kg/min for inotropic support. When the dose was titrated to 4.0 μg/kg/min in an attempt to further optimize hemodynamics, the patient developed symptomatic tachyarrhythmia, with ventricular rates reaching 180 bpm. Discontinuation of dopamine led to resolution of arrhythmia (Table 1). Based on the Naranjo Adverse Drug Reaction Probability Scale, events were classified as probable (score = 7). With appropriate management, the patient’s heart failure symptoms improved, and he was ultimately discharged in stable condition. Causality was assessed using the Naranjo Adverse Drug Reaction Probability Scale, yielding a score of 7, which categorizes an event as a “probable” adverse drug reaction.^[11]^

**Table 1:** Heart rate changes before and after dopamine infusion.

Time point	Event	Heart rate (beats/min)
Pretreatment	Before dopamine infusion	106
During treatment	During dopamine infusion	173–180
Post-treatment	1 h after dopaminediscontinuation	126
Follow-up	Following days	72–116

Heart rate significantly improved after careful dopamine titration and multidisciplinary monitoring, reflecting both the therapeutic efficacy and safety.

## 3. Discussion

The initial therapeutic decision to employ dopamine in this patient was driven by the complex hemodynamic profile of acute cardiorenal syndrome, characterized by concurrent low cardiac output (evidenced by low ejection fraction and oliguria) and significant hypotension (SBP 92 mm Hg). The pharmacological profile of dopamine, offering potential inotropic effects via β1-adrenergic stimulation at lower to intermediate doses and adjunctive vasopressor support through α-adrenergic activation at higher doses, was selected in an attempt to address both hemodynamic deficits with a single agent. This was weighed against alternatives: dobutamine, a predominantly β-agonist, carried a significant risk of exacerbating hypotension, while norepinephrine, a potent vasopressor, provides minimal direct inotropic effect. In retrospect, the subsequent development of life-threatening tachyarrhythmia demonstrates that the theoretical benefits of dopamine in such a high-risk patient were vastly outweighed by its proarrhythmic potential. This experience strongly advocates for a reevaluation of dopamine as a first-line agent in patients with a pronounced arrhythmogenic substrate, such as those with advanced age, structural heart disease, and concurrent ARNI therapy, favoring instead agents with a more favorable safety profile or a protocol-driven approach with extreme vigilance.

This case demonstrates a severe iatrogenic complication following standard-dose dopamine administration in a patient exhibiting a constellation of high-risk comorbidities, including advanced age, severe cardiorenal syndrome, and concurrent ARNI therapy. This event underscores a significant clinical knowledge deficit concerning optimal inotropic management in medically complex populations. The underlying pathophysiology warrants multifactorial analysis of several distinct domains.

Pharmacokinetic alterations secondary to severe renal impairment constitute the primary mechanistic domain, with an eGFR of 21.9 mL/min/1.73 m^2^ indicating profoundly compromised filtration capacity. Under normal physiological conditions, dopamine undergoes rapid systemic clearance, which typically becomes undetectable within minutes in individuals with intact renal function. However, severe renal disease markedly alters this pharmacokinetic profile. Impaired renal elimination substantially reduces the clearance of dopamine and its active metabolites, notably norepinephrine. Consequently, systemic accumulation of the drug occurs because of the inability of damaged kidneys to excrete these compounds effectively.^[12]^ The utilization of continuous intravenous infusion poses a particular risk in such patients. The potential for drug accumulation increases the risk of toxic plasma concentrations. This pharmacokinetic alteration potentiates excessive adrenergic receptor stimulation, particularly in the cardiovascular system. A critical consequence of this heightened stimulation is an increased propensity for cardiac tachyarrhythmias. This adverse effect is a potentially life-threatening complication associated with dopamine administration in patients with renal impairment.^[13,14]^

Beyond pharmacokinetic considerations, a confluence of structural and electrophysiological abnormalities establishes a vulnerable proarrhythmic substrate that induces sustained sympathetic nervous system activation, driving maladaptive alterations within the β-adrenergic receptor (β-AR) signaling pathway. These include the downregulation of β1-AR density and enhanced activity of G-protein-coupled receptor kinase, collectively impairing cardiac responsiveness.^[15]^ Concomitant left bundle branch block exacerbates electromechanical dyssynchrony, further compromising cardiac efficiency.^[16]^ The contributions of advanced age and diabetes mellitus independently increased the arrhythmic susceptibility. Aging promotes intrinsic alterations in myocardial ion channel function and increases interstitial fibrosis, thereby effectively lowering the threshold for arrhythmia initiation.^[17]^ Diabetic cardiomyopathy, distinct from ischemic pathology, is characterized by pro-fibrotic myocardial remodeling and mitochondrial dysfunction. These pathological features disrupt normal electrical conduction and create a pro-arrhythmic myocardial substrate.^[18]^ Within this pathologically vulnerable myocardium, a surge in circulating dopamine exerted potent pro-arrhythmic effects.^[19]^

Pharmacodynamic interactions between dopamine and neprilysin inhibition warrant specific examination, particularly when given chronic sacubitril/valsartan therapy. Sacubitril exerts its effects by inhibiting neprilysin, a ubiquitous endopeptidase responsible for degrading multiple vasoactive substrates extending beyond natriuretic peptides, including substance P, bradykinin, and angiotensin II.^[20]^ Although neprilysin does not directly catabolize catecholamines, its pharmacological inhibition significantly perturbs the neuroendocrine equilibrium.^[21]^ This complex neurohormonal shift may influence autonomic regulation, potentially altering sympathetic-parasympathetic balance. Consequently, the modified neurohumoral environment combined with direct renin-angiotensin-aldosterone system suppression may modulate sympathetic tone or adrenergic receptor sensitivity, potentially lowering the threshold for catecholamine-mediated arrhythmogenesis.^[22]^ This mechanistic interaction warrants dedicated electrophysiological investigation.^[23]^

These cumulative risk factors necessitate a critical reappraisal of inotropic selection in hemodynamically compromised patients.^[24]^ Although dopamine administration was initiated because of its combined β-adrenergic receptor agonism and α-mediated vasoconstriction, alternative pharmacotherapeutic strategies warrant consideration. Dobutamine, which exhibits relative β1-adrenergic selectivity, is a potential alternative; however, its use is frequently complicated by dose-dependent sinus tachycardia.^[25]^ Levosimendan, which operates via calcium sensitization and adenosine triphosphate-dependent potassium channel activation, provides positive inotropic support through a distinct mechanism. This agent typically demonstrates a neutral effect on myocardial oxygen consumption and a reduced pro-arrhythmic potential, although concomitant vasodilatory effects may exacerbate preexisting hypotension.^[26]^ Relevant clinical trial data demonstrated comparable mortality rates between dopamine and norepinephrine in cardiogenic shock cohorts but identified a statistically significant increase in arrhythmic events associated with dopamine administration.^[27]^ Overall, these pharmacodynamic profiles and clinical observations support the avoidance of dopamine as first-line therapy in patients with established arrhythmogenic substrates.

Collectively, these pathophysiological insights necessitate the implementation of individualized therapeutic protocols. For patients with significant arrhythmogenic risk factors, dopamine infusion should be initiated at substantially reduced rates (≤1.5 μg/kg/min) relative to conventional protocols, with cautious incremental titration mandated under continuous hemodynamic and telemetric monitoring. The implementation of a standardized safety framework requires multidisciplinary collaboration among intensivists, critical care pharmacists, and nursing personnel. Critical safeguards include electronic health record-integrated clinical decision support systems capable of automated high-risk phenotype identification, context-aware dosing recommendations aligned with renal/cardiac function, and real-time supratherapeutic dosing alerts.^[28]^ In essence, this systematic integration of comprehensive risk stratification, pharmacologically conservative initiation, and technology-enhanced vigilance constitutes an essential strategy for mitigating iatrogenic risk during vasoactive therapy.

## 4. Conclusion

Dopamine retains therapeutic utility in ADHF; however, conventional dosing regimens may precipitate life-threatening tachyarrhythmias in high-risk cohorts, particularly in elderly patients with severe cardiorenal-metabolic dysfunction. The observed toxicity likely stems from convergent pathophysiological mechanisms: pharmacokinetic accumulation secondary to impaired renal clearance and catecholamine hypersensitivity within a pathologically remodeled myocardium. These findings necessitate the abandonment of standardized dosing protocols for vulnerable populations. Optimal management requires the implementation of conservative patient-tailored infusion strategies, continuous hemodynamic and electrocardiographic surveillance, structured multidisciplinary coordination, and a critical risk-benefit assessment of alternative inotropic agents. This integrated approach maximizes the therapeutic efficacy while mitigating iatrogenic risks during hemodynamic support.

## Acknowledgments

The author expresses his sincere gratitude to the patient for his consent to publish all the clinical data in this case report.

## Author contributions

**Conceptualization:** Hui Xu.

**Data curation:** Hui Xu.

**Formal analysis:** Hui Xu.

**Investigation:** Hui Xu.

**Methodology:** Hui Xu.

**Resources:** Hui Xu.

**Validation:** Hui Xu.

**Visualization:** Hui Xu.

**Writing – original draft:** Hui Xu.

**Writing – review & editing:** Hui Xu.
